# Trials and Tribulations in Implementation of the Emergency Medicine Milestones from the Frontlines

**DOI:** 10.5811/westjem.2019.4.42061

**Published:** 2019-06-03

**Authors:** Alexander Y. Sheng

**Affiliations:** Boston University School of Medicine, Boston Medical Center, Department of Emergency Medicine, Boston, Massachusetts

## INTRODUCTION

As part of medical education’s shift toward competency-based education (CBE), the Accreditation Council for Graduate Medical Education (ACGME) announced the Milestones Project in 2008 to create an outcomes-based model of competency development. The goal was to characterize specific accomplishments or behaviors demonstrated by physician trainees as they progressed toward independent practice. Since then, multiple specialties, with emergency medicine (EM) at the forefront, have developed and incorporated competency-based assessment of residents using specialty-specific Milestones. The development of EM Milestones by the Emergency Medicine Milestone Working Group (EMMWG) has been well-described.^1^ The EMMWG identified 23 subcompetencies within the six core competencies, and within each subcompetency, five different levels of proficiency. Each level has one or more Milestones of competency to mark the level of proficiency. As part of the Next Accreditation System (NAS) implemented by the ACGME in July 2013, each Milestone subcompetency has to be reported for every resident at six-month intervals by individual residency clinical competency committees (CCC).^1^

While well-intended, methodically planned and developed, these standards have been met with various levels of exasperation and confusion by medical educators seeking to implement the new requirements.^2^ It is not my goal to push back against the Milestones approach, as it represents an iterative, dynamic process to continually advance medical education to provide safer and higher quality patient care. However, I aim to describe some frontline challenges for clinician educators attempting to implement these recommendations.

Ankel et al. predicted that “the future of CBE will require significant changes in the learning environment, resident assessment frequency, and faculty development.”^3^ Such changes have not happened in many programs, including my own. As a result, the sources of the trials and tribulations in the implementation of EM Milestones can be similarly categorized into issues with the Milestones themselves, resident assessment, direct observation, educational infrastructure, and limited resources.

### The EM Milestones

The all-encompassing nature of EM Milestones, lacking specificity to any case, disease, or context, prevents educators from reaching consensus when evaluating and assigning Milestone rankings. I frequently notice one faculty describing a trainee performing well on a Milestone behavior, ranking them highly on a particular subcompetency, while another faculty might feel differently regarding the trainee’s performance on the same subcompetency or even the same Milestone. This is because skills and behaviors in one setting may not translate to another. Trainees’ performances on tests of general constructs are known to be highly case-dependent.^4^–^6^ A resident might be fully capable of developing and narrowing down a differential diagnosis (PC4), ordering the right test (PC3), and choosing the optimal disposition (PC7) for a presentation that he or she is familiar with and be completely clueless without prior experience with such a case. Some have suggested the linking of Entrustable Professional Activities (EPA), defined as “units of physician practice in which the goal is unsupervised competent practice by a trainee” with EM Milestones.^7^ Because EPAs are based on clinical descriptions rather than individual physician descriptions, there may be less faculty development needed for Milestone subcompetency assessment.^7^ Often, EPAs are presentation or diagnosis specific, which may mitigate concerns regarding conflicting reports of trainee competency in different contexts. However, creation of EPAs for every single disease within the Model of the Clinical Practice of EM is likely too overwhelming to develop, evaluate, implement, and measure. Developers of EM Milestones would likely point out that the optimal solution is the use of multiple assessment tools to measure Milestone subcompetencies. And this leads us to my next point.

### Resident Assessment

Beeson et al. accurately anticipated the need for EM to develop multiple, valid and reliable objective measures of Milestone competency assessments.^1^ However, our field has yet to meet this challenge raised by the developers of EM Milestones.

The EM Milestones include suggested methods of evaluation that vary with subcompetency but may include direct observation, simulation, chart review, standardized patients, global ratings, multi-source feedback, and end-of-shift evaluations. However, none has been sufficiently validated to effectively evaluate a trainee’s progression through the EM Milestones. In fact, EM Milestones have been shown to possess poor inter-rater reliability between various stakeholders, such as resident self-assessment, faculty, and CCC, in various clinical settings and in simulation.^8^–^11^ Furthermore, EM Milestone ranking determined by CCC in this early stage of implementation is hardly a gold standard of comparison. Similarly, multiple assessment tools of Milestone competency failed to demonstrate significant utility.^8^,^12^ Specifically, end-of-shift evaluations of EM Milestones resulted in grade inflation compared to CCC results.^8^ A multicenter, prospective, observational study to develop a direct observation assessment of Milestones in the form of the Critical Care Direct Observation Tool demonstrated low inter-rater reliability.^12^ The authors expressed concerns for the reliability of other EM Milestone assessment tools that are currently in use.^12^

Despite mandating the semiannual review and update of the progression of EM Milestones of every resident, the EMMWG never released specific guidelines on the ideal administration and format of a CCC. Therefore, the way each CCC is run differs between residency programs.^13^ Program directors and faculty are often left to their own devices in terms of what assessment tools to use and how to assign Milestone rankings. Even though my program’s CCC uses multiple assessment tools (shift evaluations, off-service evaluations, monthly EM rotation evaluations, in-service scores, procedure, ultrasound, and simulation logs), none have been shown to be valid in the assessment of Milestone subcompetencies. After diving deeply into all available assessment data, my colleagues and I in the CCC meet in person in an attempt to build consensus in assigning Milestone rankings. Despite our best efforts, my fellow faculty and I are still left with the best “educated guess” of where each resident lies on most subcompetencies.

My department has trainees who are known to be less clinically competent but somehow consistently rank higher on EM Milestones year after year compared to their more capable peers. Much like a meta-analysis, the utility of the combined evidence depends on the strengths of the studies analyzed. The soundness and credibility of our CCC Milestone rankings leave much to be desired. My residents and faculty recognize the lack of reliability and validity in the assessment tools we use. This is demotivating to learners and educators alike, leading to less incentive for both parties to complete more assessments. The shortage of assessment data erodes faith in the Milestone evaluation process. This in turn feeds into the cycle of decreased validity and reliability of our Milestone ranking in the CCC, which further disincentivizes our residents and faculty to complete additional assessments.

### Direct Observation

The intention of using objective behaviors for EM Milestones requires direct observation to occur. Assessment of professional competence will need to be based on multiple assessment methods, each with a minimum of 8–10 observations to ensure reliable inferences.^3^ This is unrealistic for many frontline EM educators who work with limited departmental and institutional resources for faculty time for direct observation. A previous report has suggested that the overall faculty-EM resident interaction time accounts for only 20% of a resident’s time spent on a clinical shift. Direct observation time of EM residents interacting with patients by faculty in the emergency department was only 3.6% of the time.^14^ This is exacerbated by our specialty’s distinct workflow, where trainees frequently work with multiple faculty on a single shift without opportunities for sustained contact and direct observation. A monthly EM Milestone evaluation is likely low-yield since sporadic short periods of observation by multiple faculty will not illuminate a consistent picture of trainee performance. Although video precepting can be a helpful adjunct to direct observation, it is not a panacea and can be time- and resource-intensive.^15^ The same could be said of simulation programs and standardized patients.

### Educational Infrastructure

One of the advantages of CBE is that the ability to progress is not based on time. Yet in graduate medical education (GME), no system exists that allows for the residents who attain Level 4 or 5 Milestone rankings to graduate early. There’s no reward for thinking critically or to excel.^16^ Level 5 “reach” Milestones are not important goals for trainees, as EM Milestones are no longer relevant for emergency physicians after residency graduation. Academic institutions have become overly dependent on trainees to provide patient care. Any change in the rate of progression for trainees can wreak havoc on the learners’ ability to meet their service requirements and therefore disrupt the current funding model for GME.^2^ After all, “the American public is both the consumer and the financier of the United States residency training system.”^1^

Policymakers are demanding educational reform in light of healthcare inequality, cost pressures, the aging of populations, emerging diseases, and the advent of personalized medicine.^2^ Considering the need for public accountability, the main drive for the shift toward CBE has been described as political because it affects the way our government allocates resources.^16^ Quality and patient safety is an example of an area where significant resources have been allotted. However, if Milestone data are to be used to provide assurance to the public, payers, and policymakers that residency programs are providing sufficient training in targeted areas of healthcare delivery as suggested by Beeson et al.,^1^ departmental and institutional resources have to be allocated for the proper implementation and assessment of EM Milestones.

### Limited Resources

However, despite mandating the implementation of EM Milestones, resources have not been made available to individual programs for execution or medical education research to support their use. None of my fellow CCC members have been given additional protected time or administrative support to dedicate to the observation, evaluation, discussion, and assignment of EM Milestone rankings. Beeson et al. warned against the potential threats to validity of EM Milestones in the form of too few observation and bias in rankings.^1^ Given the constraints imposed by finite time and resources, it will not be possible to reliably measure more than a minute fraction of all the behaviors and scenarios that would be required to effectively evaluate a trainee’s competence. Furthermore, resources for faculty development to “ensure consistent and appropriate evaluations” deemed as important by the developers of EM Milestones have yet to materialize.^1^,^17^

Despite all this, residencies are still required by the ACGME to evaluate each resident using Milestones during CCC on a semiannual basis.^18^ The amount of time and energy, as well as faculty resources, may be inadvertently diverted away from other important educational interventions in order to facilitate this requirement. So far for my program, the efforts have not come to fruition. I hope that, through open discussion of my department’s barriers to implementation, I can encourage further dialogue to develop best practices to improve Milestone assessment and CCC administration, at my own and other programs.

## CONCLUSION

The Milestone Project is a noble, longitudinal endeavor in medical education reform that I hope will lead to improved patient outcomes. However, its implementation requires dedicated resources for research and execution at all levels. Unfortunately, those on the frontlines of EM resident education lack valid assessment tools, opportunities for direct observation, proper educational infrastructure and resources to fulfill the mandate effectively at a program level.
